# Case report: Atypical SJS/TEN-like fulminant pemphigus vulgaris: diagnostic challenges and the potential impact of anti-Dsg1 titers and CMV reactivation

**DOI:** 10.3389/fimmu.2026.1907863

**Published:** 2026-08-21

**Authors:** Alice Verdelli, Rachel Daher, Luca Sanna, Virginia Corti, Alessandro Magnatta, Simone Landini, Marta Donati, Irene Bonanni, Alberto Corrà, Valentina Ruffo di Calabria, Elena Biancamaria Mariotti, Chiara Della Bella, Lorenzo Giovannoni, Mario Milco D’Elios, Alberto Moggi Pignone, Marzia Caproni

**Affiliations:** 1 Rare Skin Diseases Unit, Section of Dermatology, Department of Health Sciences, ERN-SKIN Member, University of Florence, Florence, Italy; 2 Section of Dermatology, Department of Health Sciences, University of Florence, Florence, Italy; 3 Dermatology Department, University of Modena and Reggio Emilia, Modena, Italy; 4 Dermatology Unit, Ospedale San Bortolo, Vicenza, Italy; 5 SSD Dermatologia, Cardinal Massaia Hospital, Asti, Italy; 6 Unit of Dermatology, Azienda USL Toscana Nord Ovest, Lucca, Italy; 7 Department of Molecular and Developmental Medicine, University of Siena, Siena, Italy; 8 UOC Ricerca e Sviluppo Clinical Practice, AOU Careggi, Florence, Italy; 9 Department of Experimental and Clinical Medicine, Division of Internal Medicine, University of Florence, Careggi Hospital, Florence, Italy

**Keywords:** dermatology, immunology, intravenous immunoglobulin, paraneoplastic pemphigus, pemphigus vulgaris, SJS/TEN

## Abstract

Pemphigus vulgaris (PV) is a chronic autoimmune blistering disease characterized by intraepidermal flaccid blisters of the skin and mucous membranes. Although typically chronic and relapsing, rare fulminant variants have been described. We report a life-threatening case of PV in a previously healthy 55-year-old woman who developed a rapidly progressive, Stevens–Johnson syndrome/toxic epidermal necrolysis (SJS/TEN)-like phenotype with extensive epidermal detachment and mucocutaneous involvement. Histopathological analysis demonstrated suprabasal acantholysis, and direct immunofluorescence showed intercellular IgG and C3 deposition. Serology revealed markedly elevated anti–Desmoglein-1 (107.5 IU/mL) and modest anti–Desmoglein-3 titers (9.6 IU/mL). The clinical course was complicated by Cytomegalovirus reactivation, while malignancy screening remained negative. Treatment with rituximab and intravenous immunoglobulin led to rapid re-epithelialization and clinical stabilization. This case expands the clinical spectrum of PV and underscores diagnostic and therapeutic challenges posed by fulminant autoimmune blistering disease exhibiting overlapping features. As rat-bladder indirect immunofluorescence and extended plakin serology were not available, a paraneoplastic pemphigus (PNP)-spectrum disorder could not be formally excluded, although the clinical, histological, and serological picture strongly favored PV. We propose that markedly elevated anti-Dsg1 antibodies, complement activation, and CMV reactivation may each have contributed to this severe phenotype.

## 1 Introduction

Pemphigus disease belongs to the group of intraepithelial autoimmune bullous diseases (AIBD) affecting the skin and mucous membranes. The major subtypes include pemphigus vulgaris (PV), pemphigus foliaceus (PF), whereas paraneoplastic pemphigus (PNP), IgA pemphigus, and pemphigus herpetiformis (PH) represent the rare, atypical variants (1, 2). These disorders share overlapping immunopathogenic mechanisms but differ significantly in clinical behavior, antigenic targets, response to therapy, and prognosis. PV, the most common form, is characterized by the production of pathogenic IgG autoantibodies directed against desmosomes, primarily Desmoglein-3 (Dsg3) and Desmoglein-1 (Dsg1) (1–4). These autoantibodies disrupt keratinocyte adhesion, leading to suprabasal acantholysis and the formation of flaccid blisters (4). Pemphigus has been associated with various triggers, including malignancies, medications, and viral infections (2, 4).

While most cases of PV exhibit a chronic relapsing course responsive to immunosuppressive and/or biologic therapies, rare fulminant variants have been described (5, 6). These aggressive forms may present with rapidly progressive epidermal detachment, extensive body surface area (BSA) involvement, and clinical features that overlap with Stevens–Johnson syndrome/toxic epidermal necrolysis (SJS/TEN) or PNP. Such presentations are associated with significant diagnostic uncertainty, therapeutic resistance, and high mortality. We report here an unusual and life-threatening case of PV in an otherwise healthy middle-aged woman, characterized by rapid progression to an SJS/TEN-like phenotype, a discordant desmoglein antibody profile, and Cytomegalovirus (CMV) reactivation.

## 2 Case presentation

**Figure 1:**
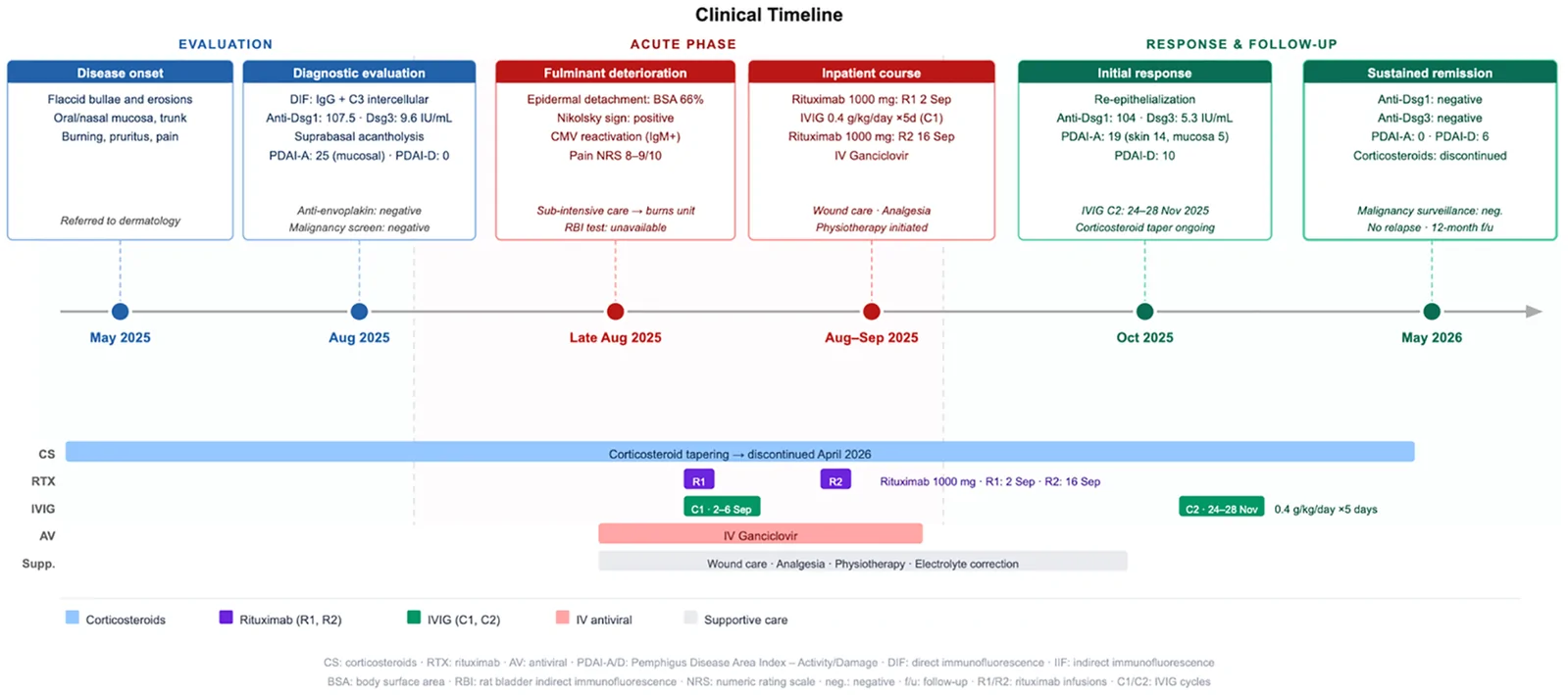
Clinical timeline. Key clinical milestones, laboratory findings, and treatment course from disease onset (May 2025) through 12-month followup (May 2026). Upper panel: clinical events at each timepoint. Lower panel: treatment timeline. CS, corticosteroids; RTX, rituximab; AV, antiviral (IVganciclovir); IVIG, intravenous immunoglobulin; R1/R2, rituximab infusions 1 and 2; C1/C2, IVIG cycles 1 and 2; PDAI-A/D, Pemphigus Disease AreaIndex — Activity/Damage; DIF, direct immunofluorescence; BSA, body surface area; RBI, rat bladder indirect immunofluorescence; NRS, numeric ratingscale; neg., negative; f/u, follow-up.

We report the case of a 55-year-old woman of Polish origin who presented with a severe, rapidly progressive mucocutaneous blistering disease (**Figure 1**). She had no personal or family history of autoimmune disease or malignancy and was otherwise healthy at baseline. She had emigrated from Poland and had lived in Italy for approximately 25 years, worked as a healthcare assistant, and was the mother of two children, one of whom has autism. Her medical history was otherwise unremarkable.

**Figure 2:**
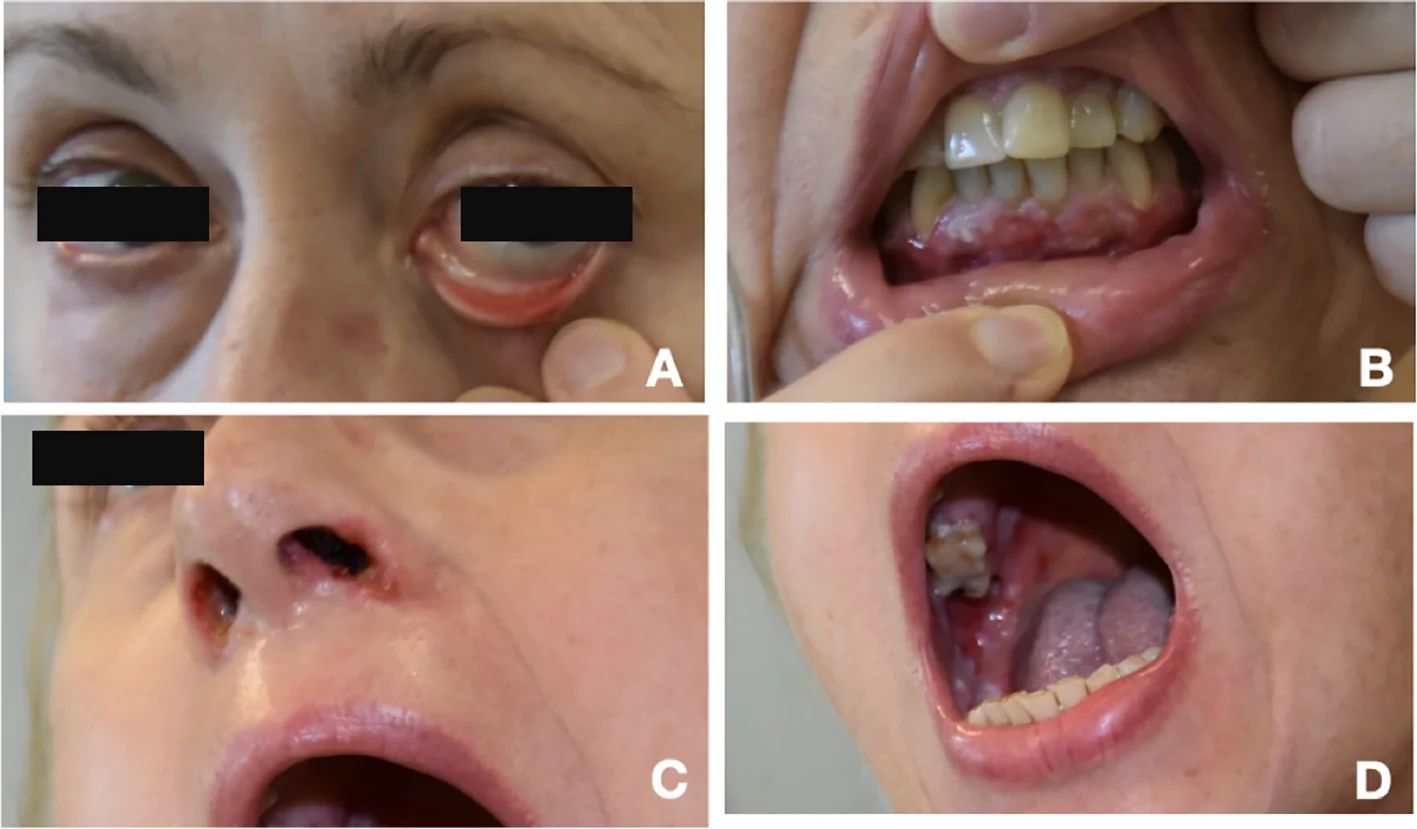
Mucosal lesions in PV. **(A)** Bilateral erosions of the inferior palpebral conjunctiva. **(B)** Erosions and desquamation of the gingival mucosa. **(C)** Erosive and ulcerative lesions of the nasal vestibule with hemorrhagic crusting. **(D)** Erosive lesions of the buccal mucosa.

**Figure 3:**
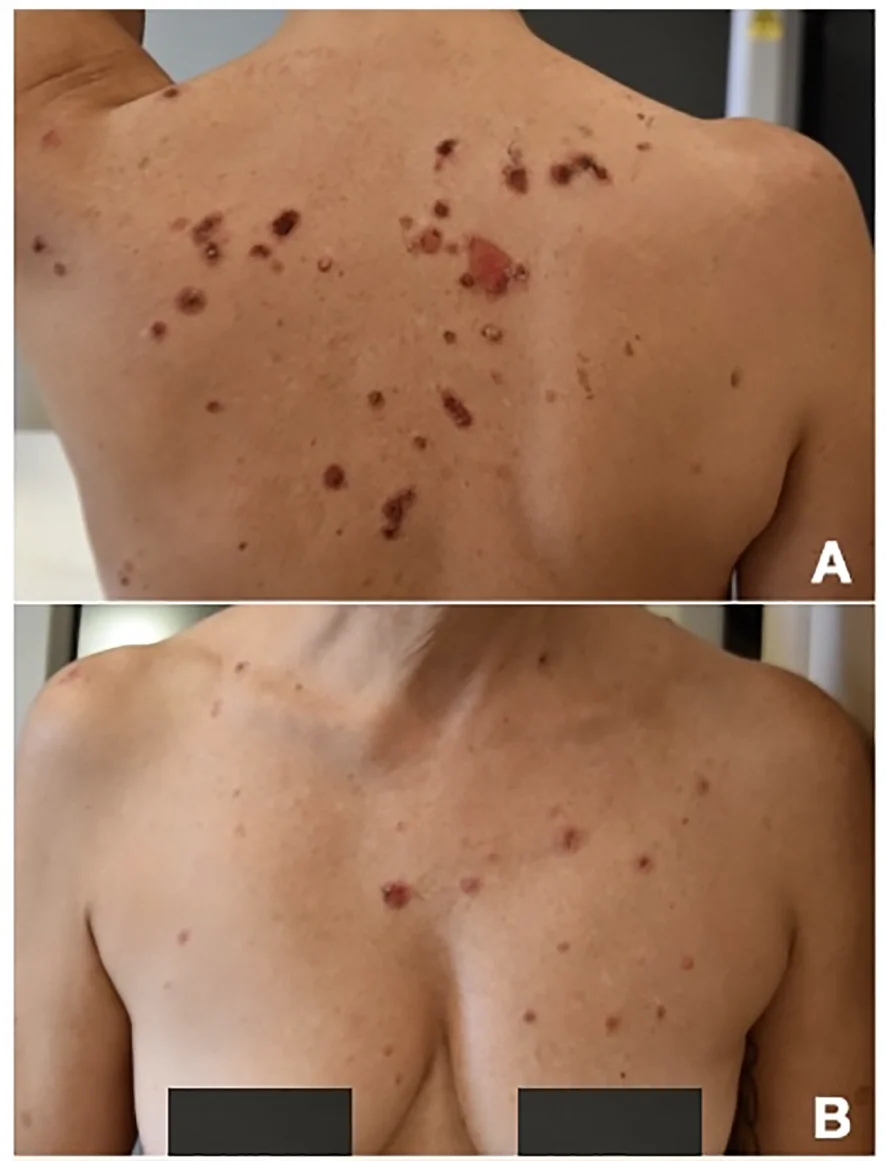
Cutaneous involvement in PV. **(A)** Multiple erosions and crusted lesions on the upper back. **(B)** Scattered erythematous erosions on the anterior chest.

The eruptions initially began in May 2025, when she developed painful flaccid bullae and erosions involving the oral and nasal mucosa (**Figure 2**) as well as the trunk, neck, and shoulders (**Figure 3**). The lesions were accompanied by intense burning and pruritus. A short course of oral prednisone was previously prescribed, with minimal clinical improvement. At her first evaluation at our dermatology unit in August 2025, she underwent indirect immunofluorescence (IIF), direct immunofluorescence (DIF) on perilesional skin, enzyme-linked immunosorbent assay (ELISA), and histopathological examination (biopsy taken from the left scapular region). Histology results displayed acantholytic dermatitis with suprabasal cleft formation, consistent with PV. DIF demonstrated intercellular deposits of Immunoglobulin (Ig) G1 (focal) and C3 within the epidermis, while IIF revealed intercellular deposits of IgG (1:80) with reactivity against Dsg1 and Dsg3. ELISA confirmed markedly elevated anti-Dsg1 (107.5 IU/mL; normal < 14) and slightly elevated anti-Dsg3 antibody levels (9.6 IU/mL; normal < 7), establishing the diagnosis of PV. Disease activity, assessed by Pemphigus Disease Area Index (PDAI), was 25. Baseline investigations included a chest radiograph, normal routine laboratory values, and negative virological screening.

The woman was started on high-dose prednisone (1 mg/kg/day, 50 mg/day) in combination with topical therapy. Rituximab infusions were scheduled for early September following completion of standard pre-treatment screening.

**Figure 4:**
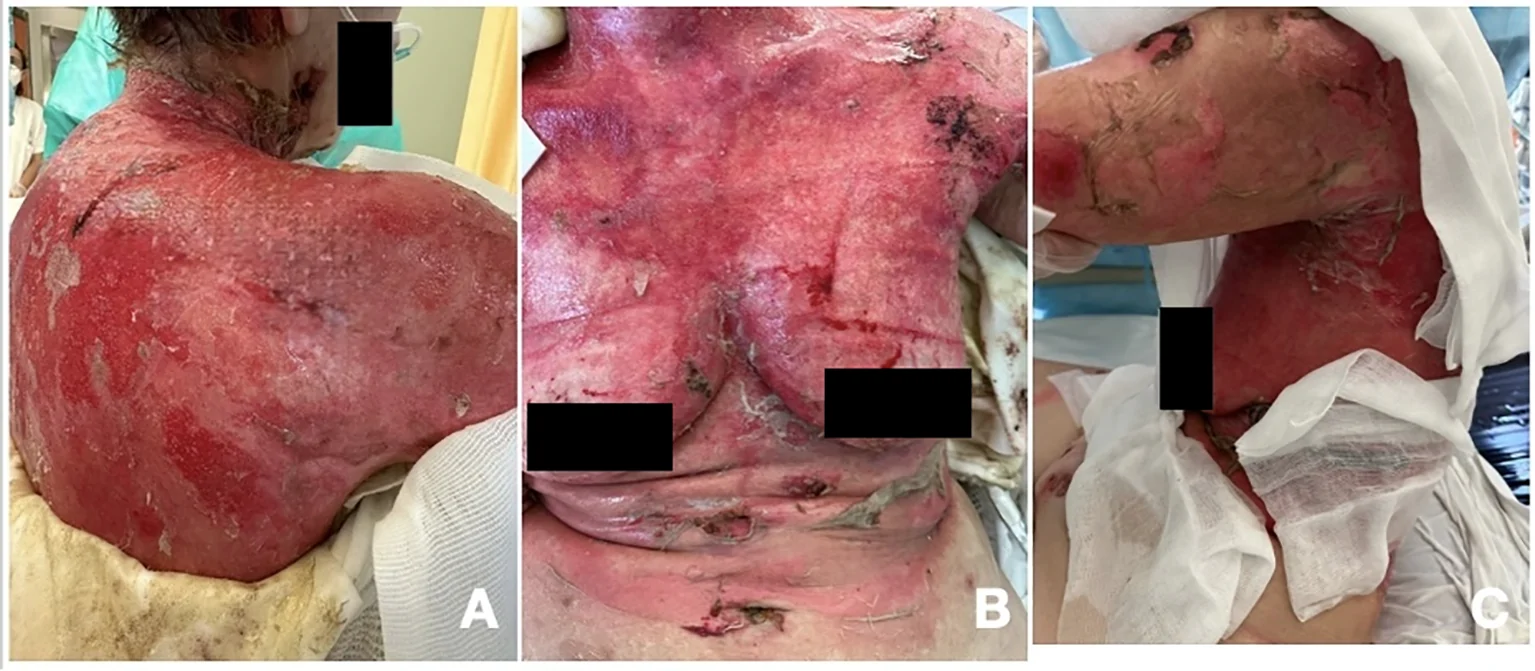
Fulminant cutaneous disease during acute deterioration. **(A–C)** Diffuse erythema, extensive epidermal detachment, and denuded skin involving the upper back, anterior trunk, and shoulders, with an estimated body surface area (BSA) involvement of approximately 66% at the time of hospitalization. Nikolsky sign was positive.

Despite systemic corticosteroid therapy, the patient experienced rapid and dramatic clinical deterioration by late August 2025, with extensive epidermal detachment involving a large BSA and progressive mucosal disease (sparing of the hands and feet) (**Figure 4**). The clinical phenotype closely resembled SJS/TEN-like PV, in the absence of systemic symptoms such as fever. She required urgent hospitalization and was transferred to a sub-intensive care setting. Body surface area involvement at admission was estimated at approximately 66% using the rule of nines by several experienced dermatologists, and disease activity was scored using the Pemphigus Disease Area Index (PDAI) by the attending dermatology team.

During hospitalization, microbiological investigations excluded bacterial superinfection but revealed CMV reactivation (positive CMV IgM). An extensive malignancy workup was negative and included chest radiography, mammography, abdominal ultrasonography, fecal occult blood testing, and cervical cancer screening. Anti-envoplakin antibody testing was negative (0.1 IU/mL). Indirect immunofluorescence on rat bladder epithelium (RBI test), the most specific assay for PNP, was unfortunately not available in our laboratory at the time. CMV reactivation was identified by positive CMV IgM serology during hospitalization; quantitative CMV-DNA (viral load) testing was not performed. Beyond the negative anti-envoplakin result, extended plakin antibody profiling (e.g., periplakin and desmoplakin) was likewise unavailable.

The patient received intravenous ganciclovir, broad-spectrum antibiotics, supportive care, and specialized wound management. During hospitalization, the patient reported severe pain from her skin lesions, rated as 8–9/10 on the Numerical Rating Scale (NRS), and required opioid analgesia and sedation during dressing changes. The patient also developed marked polyuria associated with hypokalemia and hyponatremia, necessitating frequent electrolyte replacement and the implementation of a high-sodium diet.

**Figure 5:**
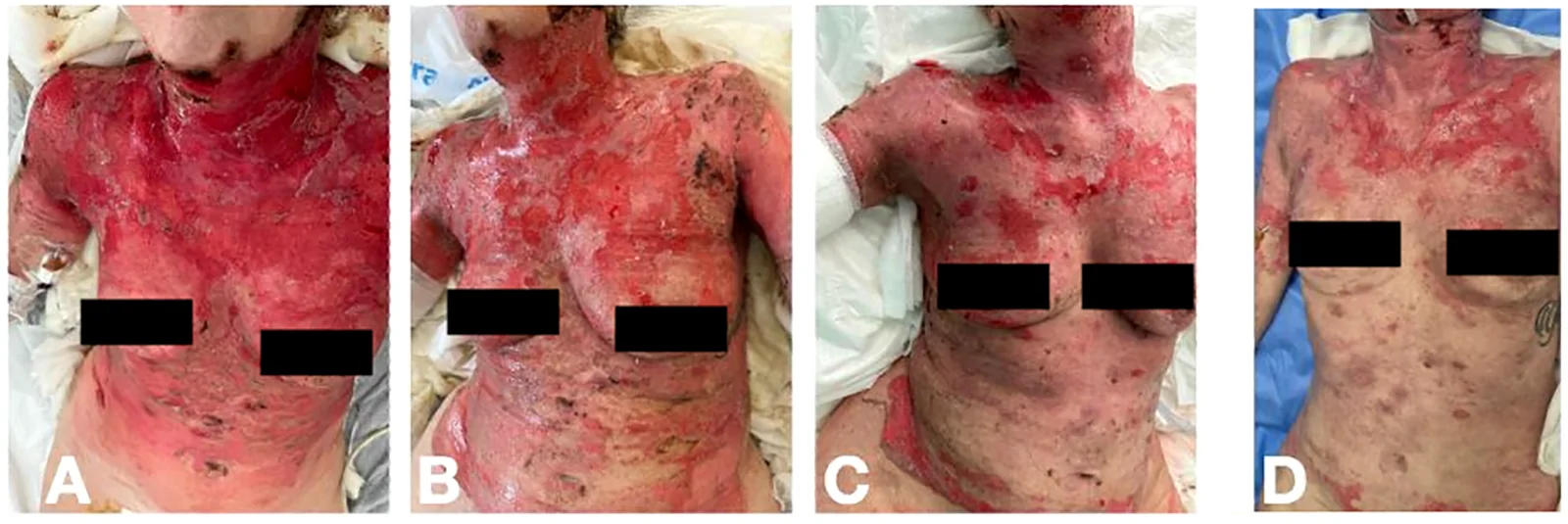
Cutaneous response to intravenous immunoglobulin (IVIG) therapy. **(A–D)** Sequential clinical images showing progressive re-epithelialization and reduction of erythema and erosions following IVIG therapy: **(A)** baseline (T0, prior to first IVIG infusion), **(B)** day 2 (T2), **(C)** day 4 (T4), and **(D)** day 6 (T6) after treatment initiation.

To address the PV, given the severity and steroid-refractory course, the patient was treated with rituximab (1000 mg intravenously, two doses 15 days apart) combined with intravenous immunoglobulins (IVIG; 0.4 g/kg/day for 5 consecutive days). Early re-epithelialization was observed within days of IVIG initiation (**Figure 5**). Due to extensive cutaneous involvement, she was transferred to a specialized burns unit for advanced skin barrier management and recovery. Because the rapid deterioration precluded the originally planned outpatient rituximab, B-cell-directed therapy was escalated urgently in the inpatient setting: the first rituximab infusion was administered and was immediately followed by IVIG to provide rapid immunomodulation during the interval before rituximab-induced B-cell depletion.

At follow-up in September and October 2025, she showed gradual cutaneous re-epithelialization and clinical stabilization, although with residual post-inflammatory dyspigmentation. Serologic monitoring revealed persistently elevated anti-Dsg1 antibodies (104 IU/mL) with negative anti-Dsg3 antibodies (5.3 IU/mL). At the October 2025 evaluation, her PDAI Activity Score was 19 (skin 14, mucosa 5), with a Damage Score of 10 reflecting post-inflammatory changes. Given the ongoing disease activity, a second cycle of IVIG was scheduled for November 2025, alongside continued low-dose corticosteroid tapering and close multidisciplinary follow-up. Serial anti-Dsg1 titers declined in parallel with clinical improvement (107.5 IU/mL at admission, 104 IU/mL in October 2025, and negative at the most recent follow-up), with progressive reduction in body surface area involvement and re-epithelialization.

At 12-month follow-up in May 2026, the patient showed complete clinical remission (PDAI Activity Score 0), with no active cutaneous or mucosal disease. She remains under close dermatological and oncological surveillance, with repeat malignancy screening remaining negative.

## 3 Discussion

This case demonstrates an atypical and life-threatening disease course of PV in an otherwise healthy middle-aged woman. The clinical behavior of the disease challenged conventional boundaries between classic PV, PNP, and fulminant PV phenotypes.

At disease onset, the patient presented clinical and histopathological features consistent with classic PV. The subsequent course was marked by a fulminant deterioration characterized by extensive epidermal detachment of a large BSA and severe mucocutaneous involvement, clinically consistent with an SJS/TEN-like PV phenotype.

PNP is a rare but severe autoimmune blistering condition that is almost always associated with an underlying neoplasm, most often lymphoproliferative (6). It is characterized by autoantibodies directed against multiple plakin-family antigens, including envoplakin, periplakin, desmoplakin, BP230, and A2ML1, with indirect immunofluorescence on RBI representing a highly specific diagnostic assay for PNP. In our patient, anti-envoplakin antibodies were negative, comprehensive malignancy screening was unremarkable, and chest radiography did not reveal features of bronchiolitis obliterans, a complication characteristic of PNP-associated pulmonary involvement. However, we acknowledge that RBI testing was not performed, representing a limitation that precludes complete serological exclusion. Taken together, the available clinical, immunological, and oncological data make PNP unlikely in this patient, although not definitively excluded.

At 12-month follow-up (May 2026), the patient remains in complete clinical and serological remission (PDAI-A 0; anti-desmoglein antibodies negative), and repeated oncological surveillance has remained negative. This is clinically relevant, as approximately two-thirds of PNP patients present with a known malignancy at or before disease onset (20). The absence of detectable neoplasia at 12 months, together with sustained remission following B-cell–directed therapy, further supports the diagnosis of severe SJS/TEN-like pemphigus vulgaris over a PNP-spectrum disorder, although continued long-term surveillance remains warranted in line with current EADV S2k guidelines (7, 8).

SJS/TEN-like presentations in the pemphigus spectrum are rare and have been described predominantly in the context of PNP, where rapid epidermal detachment, mucosal involvement, and corticosteroid refractoriness have led to its proposal as a distinct clinical subtype (5, 6). True SJS/TEN-like PV — with classic pemphigus serology and no drug trigger — is exceedingly rare. The clinical evolution of our patient closely mirrors this phenotype. The distinction from true SJS/TEN rests on three converging lines of evidence: the absence of a drug trigger; histopathological demonstration of suprabasal acantholysis, fundamentally distinct from the full-thickness epidermal necrosis and keratinocyte apoptosis of SJS/TEN; and direct immunofluorescence showing intercellular IgG and C3 deposition, incompatible with SJS/TEN (**Table 1****).** The present case most closely aligns with SJS/TEN-like PV: serological and histopathological findings are consistent with PV, while the incomplete autoantibody profile means a PNP-spectrum disorder cannot be fully excluded. Regardless of nosological classification, this case demonstrates that severe, SJS/TEN-like epidermal detachment can occur within the pemphigus spectrum and warrants early B-cell–directed therapy.

**Table 1:** Key distinguishing features between Pemphigus Vulgaris (PV), Stevens–Johnson Syndrome/Toxic Epidermal Necrolysis (SJS/TEN), and Paraneoplastic Pemphigus (PNP), with mapping of the present case.

Feature	Classic PV	SJS/TEN	PNP
Skin histology	Suprabasal acantholysis	Full-thickness necrosis/apoptosis	Suprabasal acantholysis + interface dermatitis
Direct IF	Intercellular IgG + C3	Negative or non-specific	Intercellular + BMZ deposits
Autoantibodies	Anti-Dsg1, Anti-Dsg3	None (drug/immune-mediated)	Anti-plakin (envoplakin, periplakin, desmoplakin)
Drug trigger	Possible (not required)	Usually present	Absent
Malignancy	Not required	Not required	Almost always present
Rat bladder IF (RBI)	Negative	Negative	Positive (highly specific diagnostic test for PNP)
Pulmonary involvement	Absent	Absent	Present in 30–40% of cases
Present case	Suprabasal acantholysis, IgG intercellular DIF	Absent drug trigger; acantholysis, not necrosis	Anti-envoplakin negative; no malignancy detected; RBI not performed (limitation)

Another potential contributor to the observed clinical deterioration is viral-driven immune dysregulation. During hospitalization, the patient demonstrated evidence of CMV reactivation. An association between pemphigus and viral infections, particularly CMV, has been reported, although a causal relationship remains unproven (2, 9, 10). Three non-mutually exclusive mechanisms may be considered: CMV as a trigger of autoimmune dysregulation (e.g., via molecular mimicry or bystander activation); as a disease amplifier exacerbating epidermal injury in established disease; or as a consequence of iatrogenic immunosuppression from prolonged corticosteroid use. In the present case, the relative contribution of these mechanisms cannot be determined. Antiviral therapy with ganciclovir was initiated promptly, followed by gradual clinical improvement during hospitalization. Notably, CMV IgM positivity emerged only during hospitalization, after high-dose corticosteroids had been started and once cutaneous deterioration was already advanced, a temporal sequence more consistent with CMV acting as an amplifier of established disease or as a consequence of immunosuppression than as an initiating trigger. As the diagnosis rested on CMV IgM serology without quantitative CMV-DNA confirmation, any pathogenic role should be interpreted with appropriate caution.

The immunological profile adds a further layer of diagnostic complexity and may help explain the SJS/TEN-like cutaneous phenotype. The markedly elevated anti-Dsg1 titer (107.5 IU/mL) in the presence of only modest anti-Dsg3 (9.6 IU/mL) represents a discordant sero-clinical pattern in the context of mucosal involvement. Critically, Dsg1 is the predominant desmoglein isoform in the superficial and mid-epidermis and is particularly abundant in areas of high mechanical stress. Its antibody-mediated disruption leads to loss of keratinocyte cohesion within the upper epidermal layers, resulting in sheet-like epidermal fragility and detachment (11, 12). Although this mechanism is immunologically distinct from the keratinocyte apoptosis and necrosis that characterize SJS/TEN, it may produce a clinically indistinguishable phenotype of widespread epidermal loss. This provides a plausible mechanistic basis for the SJS/TEN-like presentation observed in our patient. Consistent with prior studies, including Harman et al. and subsequent prospective analyses, anti-Dsg1 titers correlate with the extent and severity of cutaneous involvement (12, 13). In this context, the markedly elevated Dsg1 level observed in our patient may have contributed to the severity of epidermal detachment and the SJS/TEN-like clinical appearance. A further immunological contributor may be complement-mediated injury. Direct immunofluorescence in our patient demonstrated intercellular IgG1 together with C3 deposition; as IgG1 is a complement-fixing IgG subclass, this pattern suggests that local complement activation may have amplified antibody-mediated acantholysis. Complement-driven keratinocyte injury could therefore represent an additional mechanism underlying the fulminant, SJS/TEN-like phenotype, and may also help explain the rapid clinical response to intravenous immunoglobulin, which exerts part of its therapeutic effect through complement modulation. Mechanistically, IgG1 and IgG3, unlike IgG4, activate the classical complement pathway; the presence of intercellular IgG1 together with C3 therefore raises the possibility that classical-pathway complement activation contributed to keratinocyte injury, in addition to the direct, largely complement-independent steric and intracellular signaling mechanisms that predominate in pemphigus acantholysis (10). This interpretation is consistent with recent clinical data showing associations between altered immunoglobulin subclasses and elevated complement levels and disease severity in pemphigus (21), bolstering the plausibility that complement activation amplifies antibody-mediated acantholysis in fulminant presentations. In the absence of complement functional studies this contribution remains to be confirmed, but the intercellular IgG1 and C3 findings make it a biologically plausible amplifier of disease severity.

The management of this patient posed significant challenges due to the severity of cutaneous involvement, the high risk of infectious complications, and the absence of specific therapeutic guidelines for fulminant pemphigus. Systemic corticosteroids alone were insufficient to control disease, and prolonged high-dose therapy was considered unsafe given the infectious risk. IVIG was therefore added as adjunctive therapy, resulting in rapid re-epithelialization and a sustained clinical response.

This approach is supported by prior evidence demonstrating the value of combined rituximab and IVIG in severe or rapidly progressive pemphigus. Ahmed et al. showed that rituximab followed by monthly IVIG cycles induced complete remission in treatment-refractory PV (14). More recent reports have confirmed the synergistic benefit of this regimen in high-risk pemphigus variants, with IVIG providing immediate immunomodulation and epidermal stabilization while rituximab establishes longer-term B-cell depletion (15–17). IVIG is typically administered in monthly cycles for 3 to 6 months, with clinical improvement often occurring after the first or second course (15). The rapidity of re-epithelialization should not be attributed to any single agent. Beyond complement modulation, IVIG acts through blockade and saturation of neonatal and activating Fc-gamma receptors, accelerated clearance of pathogenic IgG, and neutralization of autoantibodies by anti-idiotypic antibodies, alongside broad immunomodulatory effects (14, 15). Accordingly, the favorable outcome in our patient is best understood as the result of combined therapy (systemic corticosteroids, rituximab, IVIG, antiviral treatment, and intensive supportive care) rather than of IVIG alone.

Supportive management played a crucial role in recovery. Repeated sedated dressing changes were required to manage pain from extensive epidermal detachment, a practice described in other cases of severe epidermal loss when conventional analgesia is insufficient (18). Both prolonged sedation and strong opioid analgesia carry important drawbacks — including respiratory depression, hypotension, reduced bowel motility, and impaired rehabilitation — and must be applied judiciously (18, 19). The patient also required intensive fluid and electrolyte correction for significant polyuria, hyponatremia, and hypokalemia. Early physiotherapy was essential, as prolonged immobilization resulted in marked muscle wasting and functional decline. These interventions underscore that the management of fulminant pemphigus extends well beyond immunosuppression and demands coordinated multidisciplinary care.

Several limitations of this case report warrant acknowledgement. As with any single-center case report, generalizability is inherently limited. The temporary unavailability of rat bladder indirect immunofluorescence at our center represents the main serological limitation, as this assay would have further strengthened PNP exclusion. The role of CMV in disease exacerbation, while biologically plausible, cannot be established as causal from a single case. These considerations reinforce the value of comprehensive immunological workup and long-term multidisciplinary follow-up in all atypical or severe presentations of autoimmune blistering diseases. CMV reactivation was documented by IgM serology without quantitative CMV-DNA confirmation, further limiting inferences about its pathogenic role. Extended plakin antibody profiling was likewise unavailable, so a PNP-spectrum disorder cannot be entirely excluded. In addition, no repeat skin biopsy was obtained during the fulminant phase to confirm ongoing acantholysis, although serial immunofluorescence studies at follow-up were consistent with disease inactivity. As these mechanistic interpretations derive from a single case, they are intended as hypothesis-generating and warrant confirmation in larger cohorts; they nonetheless build on well-characterized disease mechanisms and the patient’s own immunopathological findings.

In summary, this case expands the recognized clinical spectrum of PV by illustrating a fulminant, SJS/TEN-like PV disease course in the absence of traditional risk factors. It highlights the potential role of high anti-Dsg1 titers and viral reactivation as modifiers of disease severity, and the diagnostic difficulty of distinguishing severe PV from PNP-spectrum disease when autoantibody profiling is incomplete. It further supports early B-cell–directed therapy in steroid-refractory, rapidly progressive pemphigus, and reinforces the need for long-term oncological and virological surveillance in atypical or severe presentations. While these mechanistic contributions warrant confirmation in larger series, the case underscores the importance of recognizing fulminant, SJS/TEN-like PV and of initiating early B-cell-directed therapy in steroid-refractory disease.

## 4 Patient perspective

Throughout the course of her illness, the patient experienced significant physical and emotional distress. The extensive skin detachment, sometimes requiring sedation, was described as extremely painful and frightening, profoundly limiting her autonomy and quality of life. Prolonged hospitalization and immobilization led to marked muscle weakness, necessitating physiotherapy and assisted ambulation. The development of electrolyte disturbances and the need for strict dietary adjustments further contributed to her sense of vulnerability. In addition, she experienced symptoms of depressed mood, emotional exhaustion, and fear concerning the unpredictability of her condition, which required psychological support. Despite these challenges, gradual clinical improvement and functional recovery provided her with motivation to actively engage in rehabilitation and long-term care.

## Data Availability

The original contributions presented in the study are included in the article. Further inquiries can be directed to the corresponding author.
